# Supplementary figures and images for: Impacts of land use and invasive species on native avifauna of Mo’orea, French Polynesia
Source: PeerJ. 2017 Sep 15;5:e3761. doi: 10.7717/peerj.3761 (PMC5602700; doi:10.7717/peerj.3761)

# Common Myna

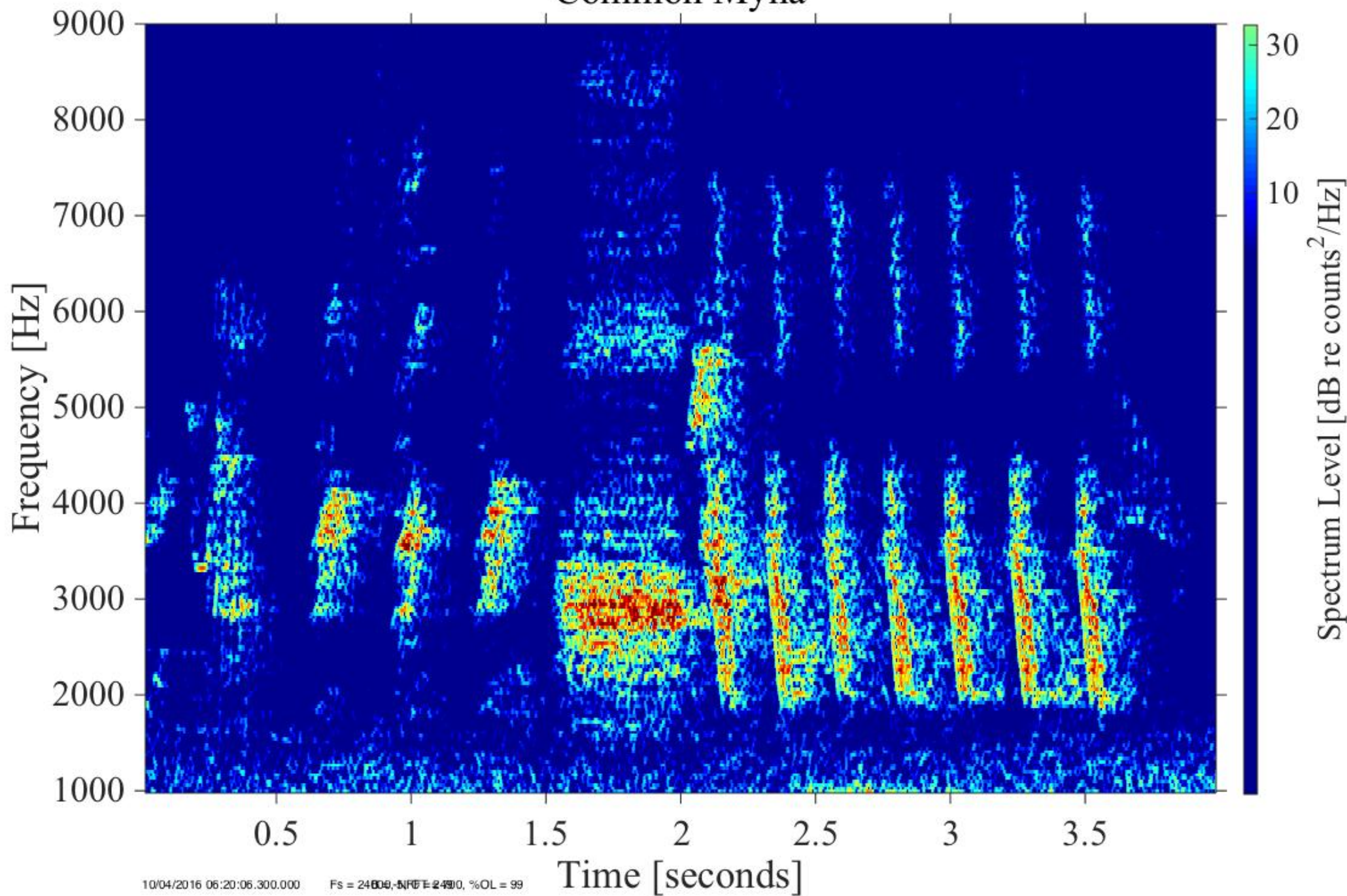

10/04/2016 06:20:06.300.000 Fs = 24000, NFFT = 2400, %OL = 99

Supplement: Supplemental Information 2 — This spectrogram shows a 4 s window with a common myna call. The x-axis shows time in seconds. The y-axis shows frequency in Hz and spectrum level in dB re counts2/Hz. [file peerj-05-3761-s002.pdf]

# Common Waxbill

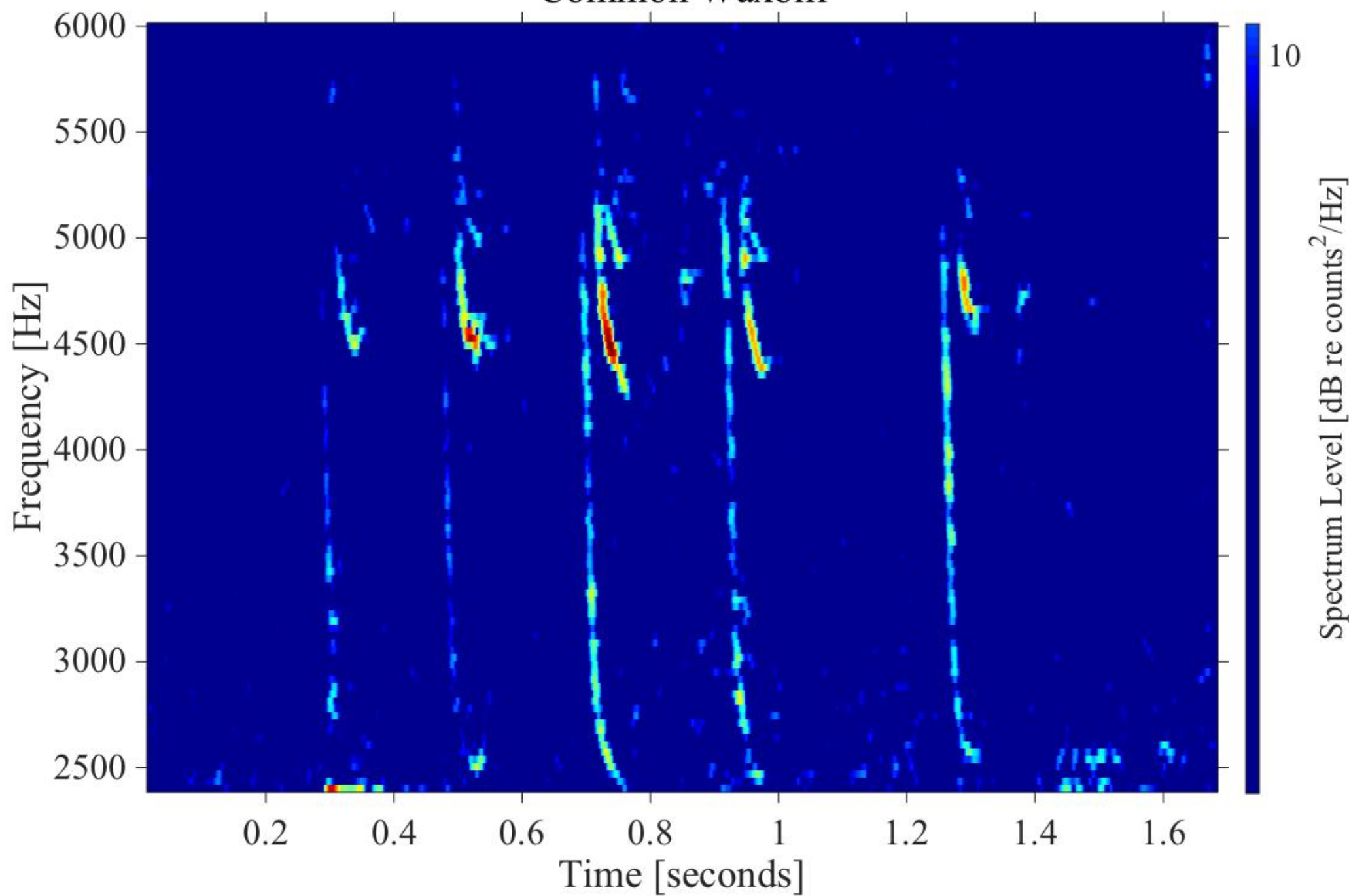

Supplement: Supplemental Information 3 — This spectrogram shows a 1.6 s window with a five common waxbill darts. The x-axis shows time in seconds. The y-axis shows frequency in Hz and spectrum level in dB re counts2/Hz. [file peerj-05-3761-s003.pdf]

# Red-browed Firetail

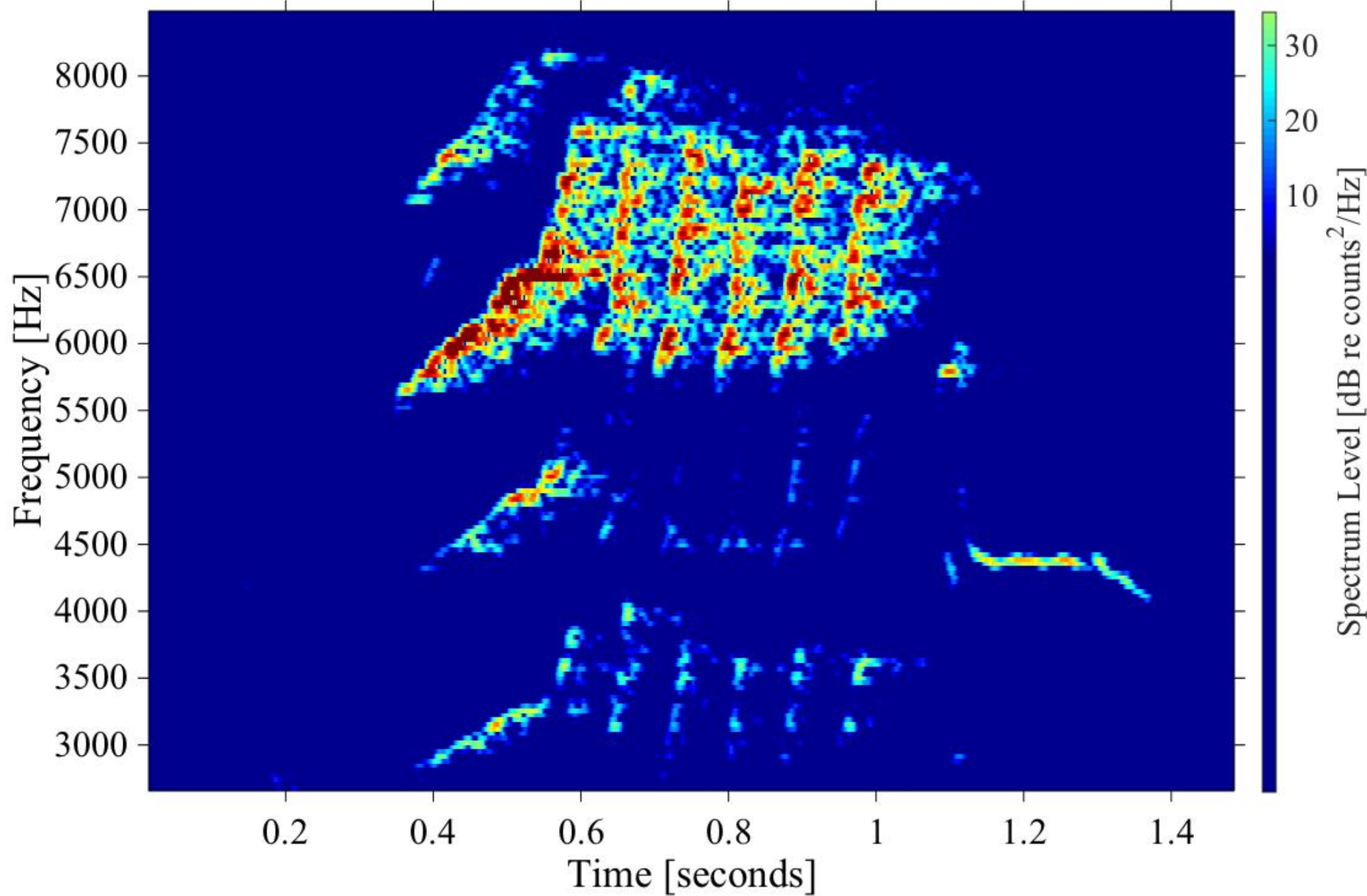

Supplement: Supplemental Information 5 — This spectrogram shows a 1.5 s window with one red-browed firetail upsweep trill. The x-axis shows time in seconds. The y-axis shows frequency in Hz and spectrum level in dB re counts2/Hz. [file peerj-05-3761-s005.pdf]

# Grey-green Fruit Dove

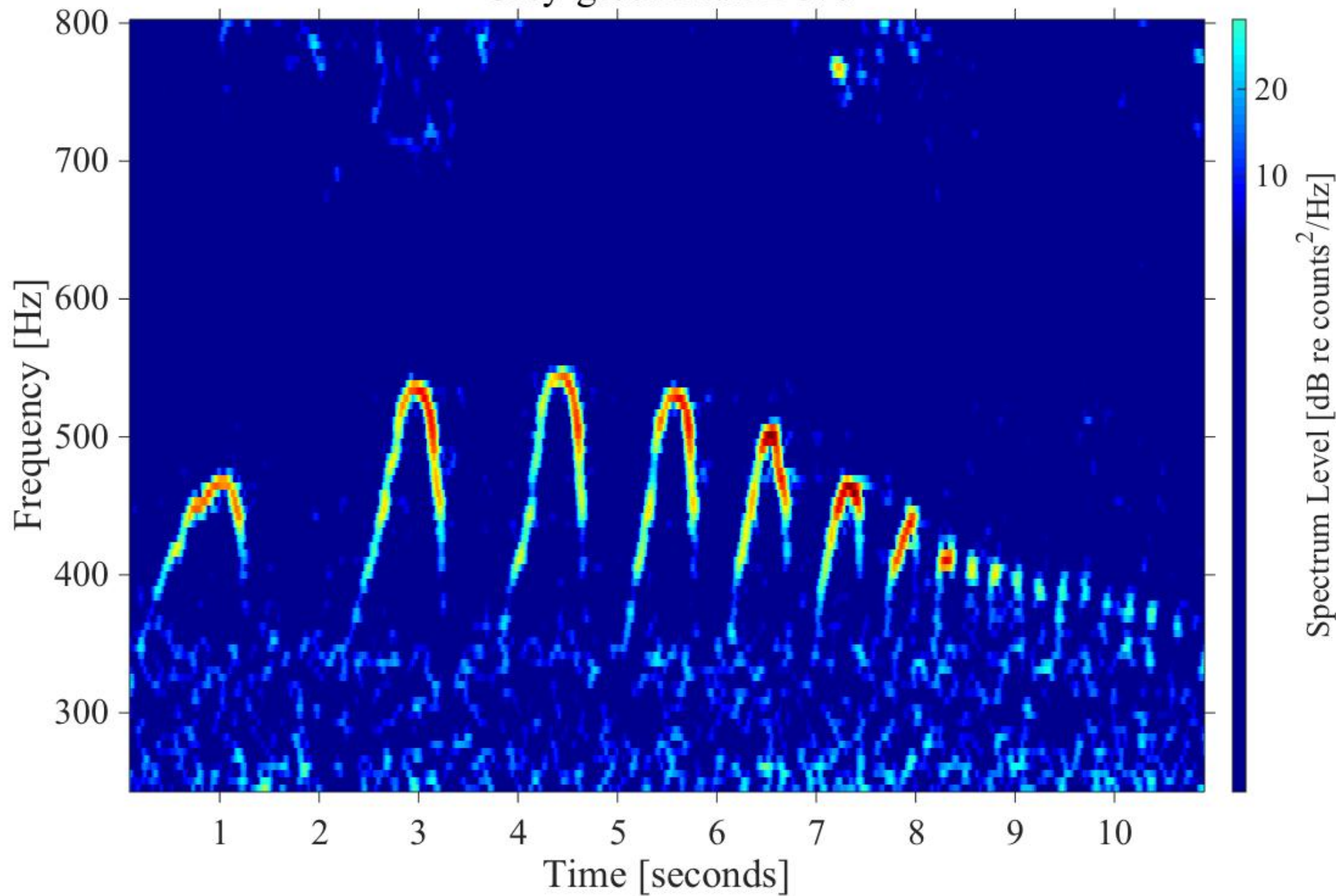

Supplement: Supplemental Information 6 — This spectrogram shows a 12 s window with one coo-train from the grey-green fruit dove. This is one example of what a grey-green fruit dove coo-train can look like. The x-axis shows time in seconds. The y-axis shows frequency in Hz and spectrum level in dB re counts2/Hz. [file peerj-05-3761-s006.pdf]

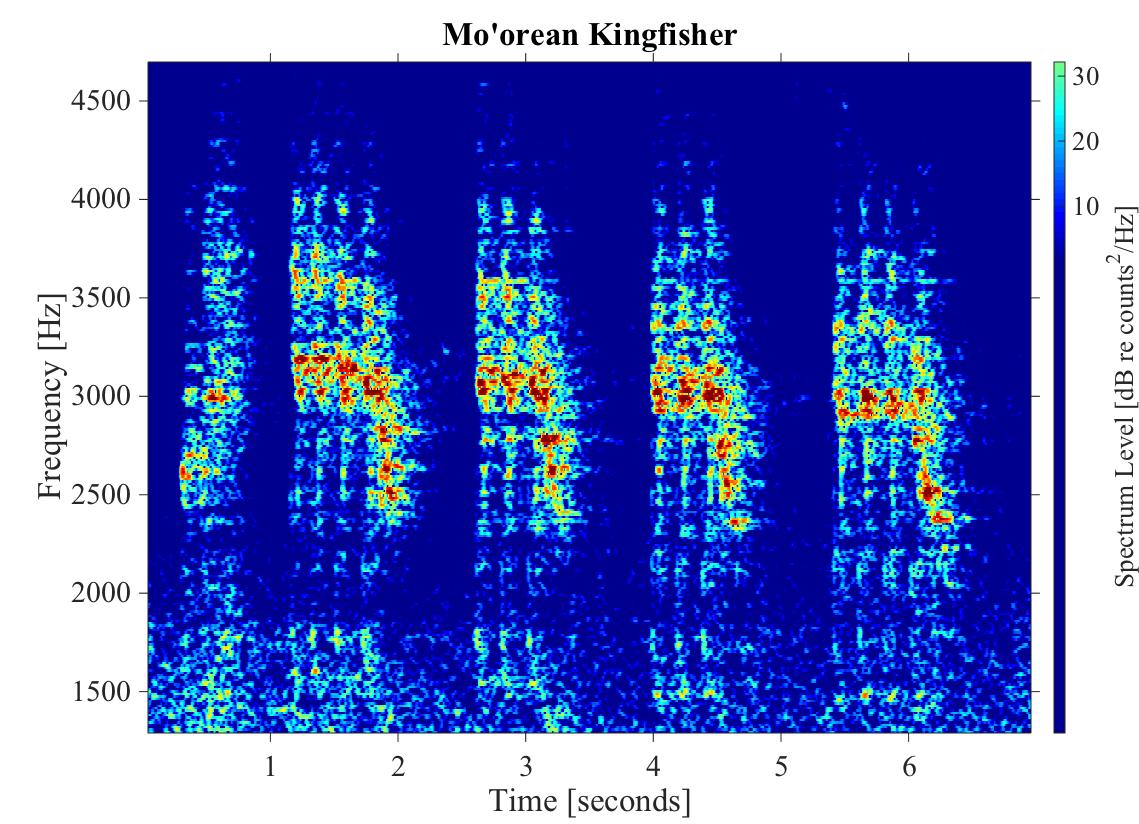

Supplement: Supplemental Information 7 — This spectrogram shows a 7 s window of one shuddering klew from a Mo’orean kingfisher. The x-axis shows time in seconds. The y-axis shows frequency in Hz and spectrum level in dB re counts2/Hz. [file peerj-05-3761-s007.jpg]

# Red Jungle Fowl

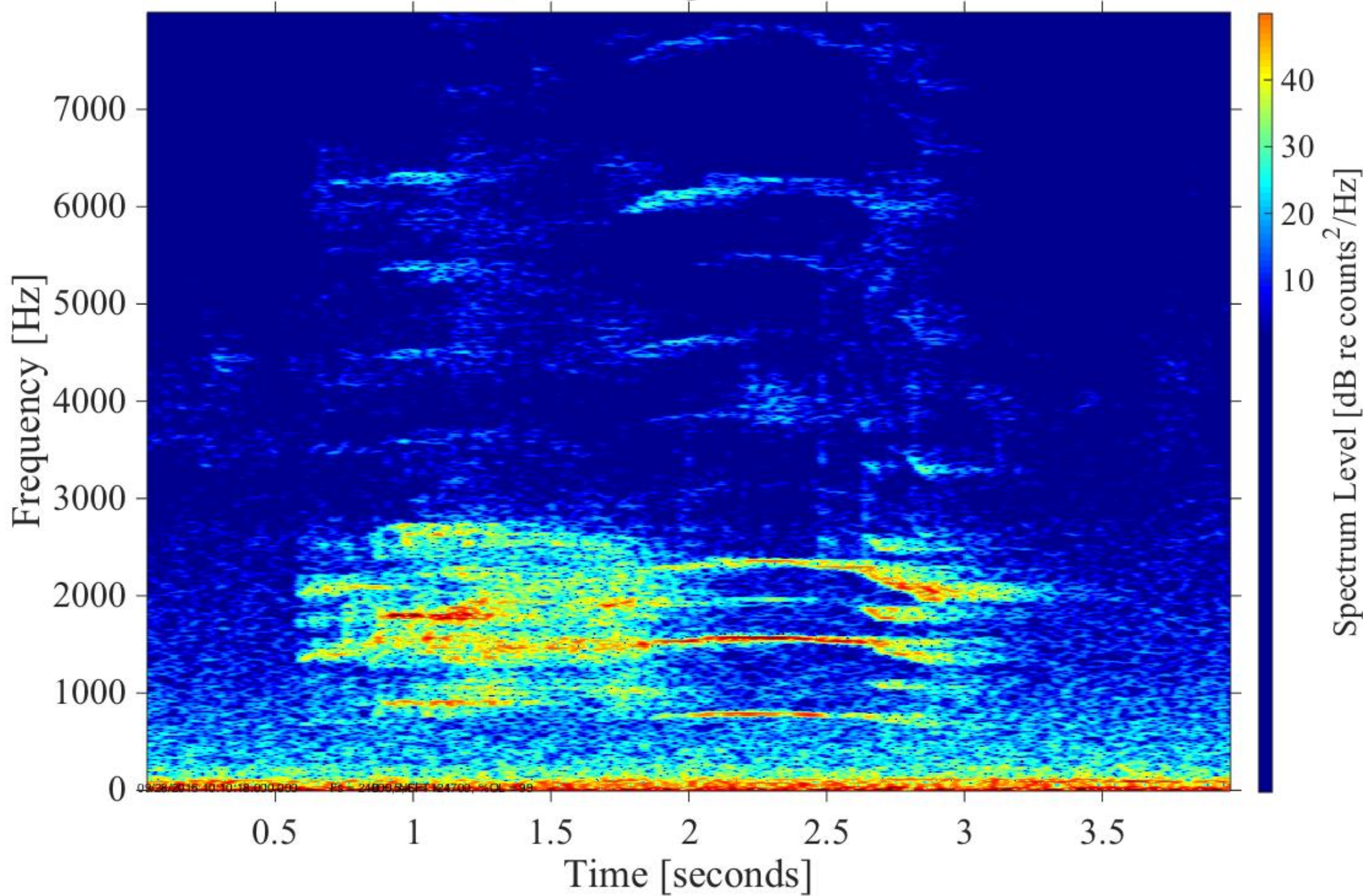

Supplement: Supplemental Information 8 — This spectrogram shows a 4 s window of one screech from a red jungle fowl. The x-axis shows time in seconds. The y-axis shows frequency in Hz and spectrum level in dB re counts2/Hz. [file peerj-05-3761-s008.pdf]

# Red-vented Bulbul

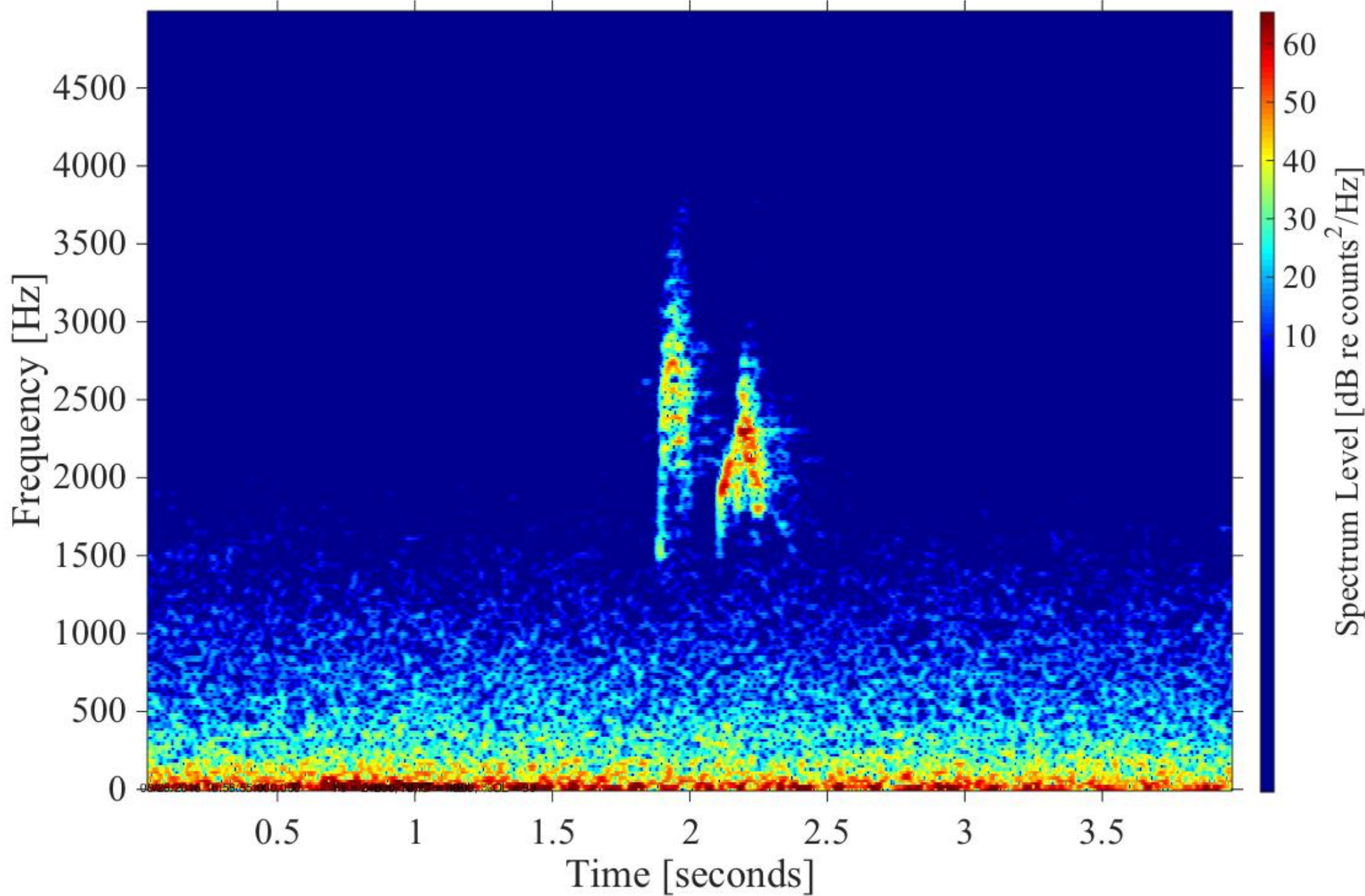

Supplement: Supplemental Information 9 — This spectrogram shows a 4 s window of a two-toned call from a red-vented bulbul. The x-axis shows time in seconds. The y-axis shows frequency in Hz and spectrum level in dB re counts2/Hz. [file peerj-05-3761-s009.pdf]

# Silvereye

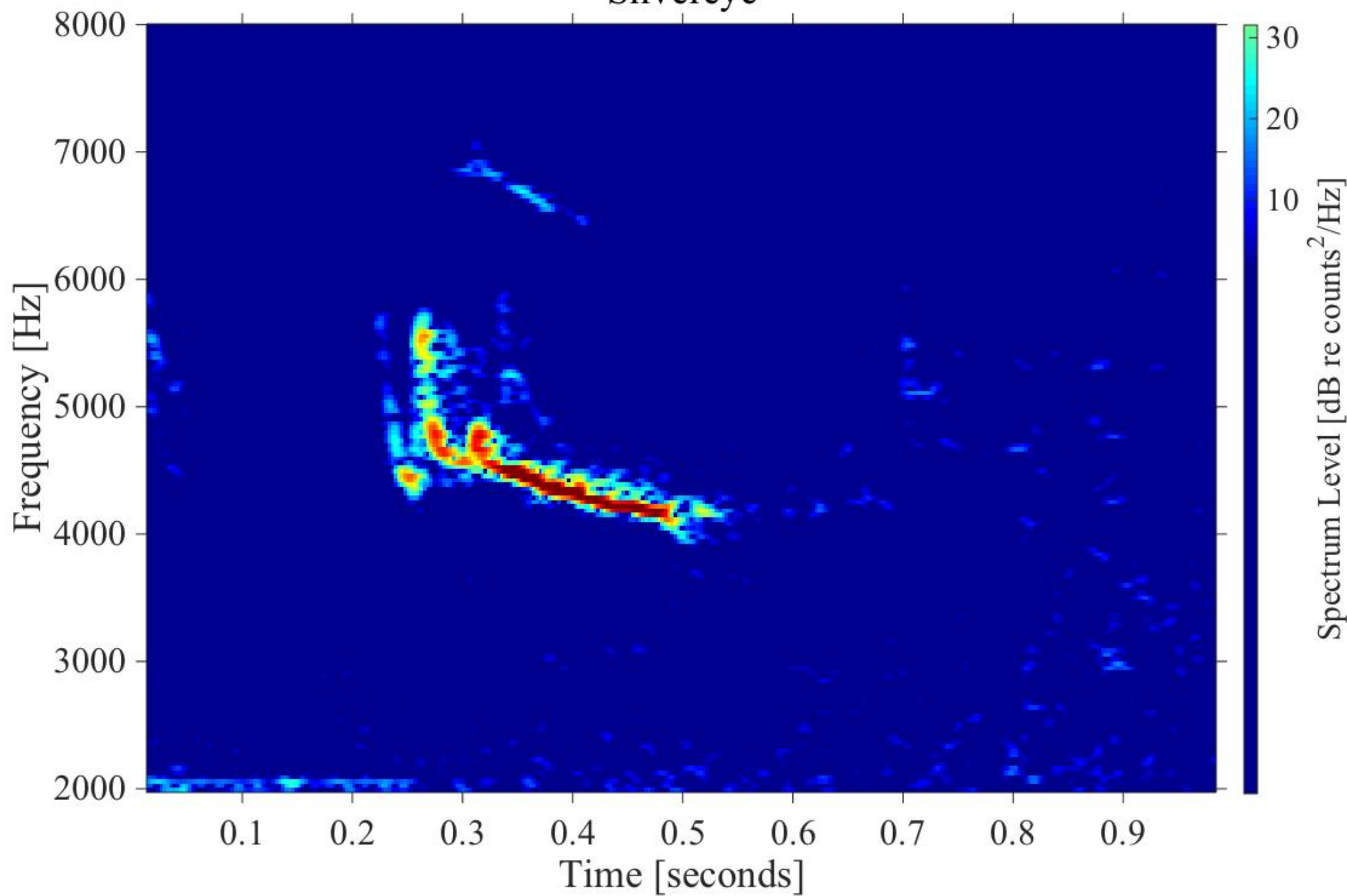

Supplement: Supplemental Information 10 — This spectrogram shows a 1 s window of one downsweep from a silvereye. The x-axis shows time in seconds. The y-axis shows frequency in Hz and spectrum level in dB re counts2/Hz. [file peerj-05-3761-s010.pdf]

# Zebra Dove

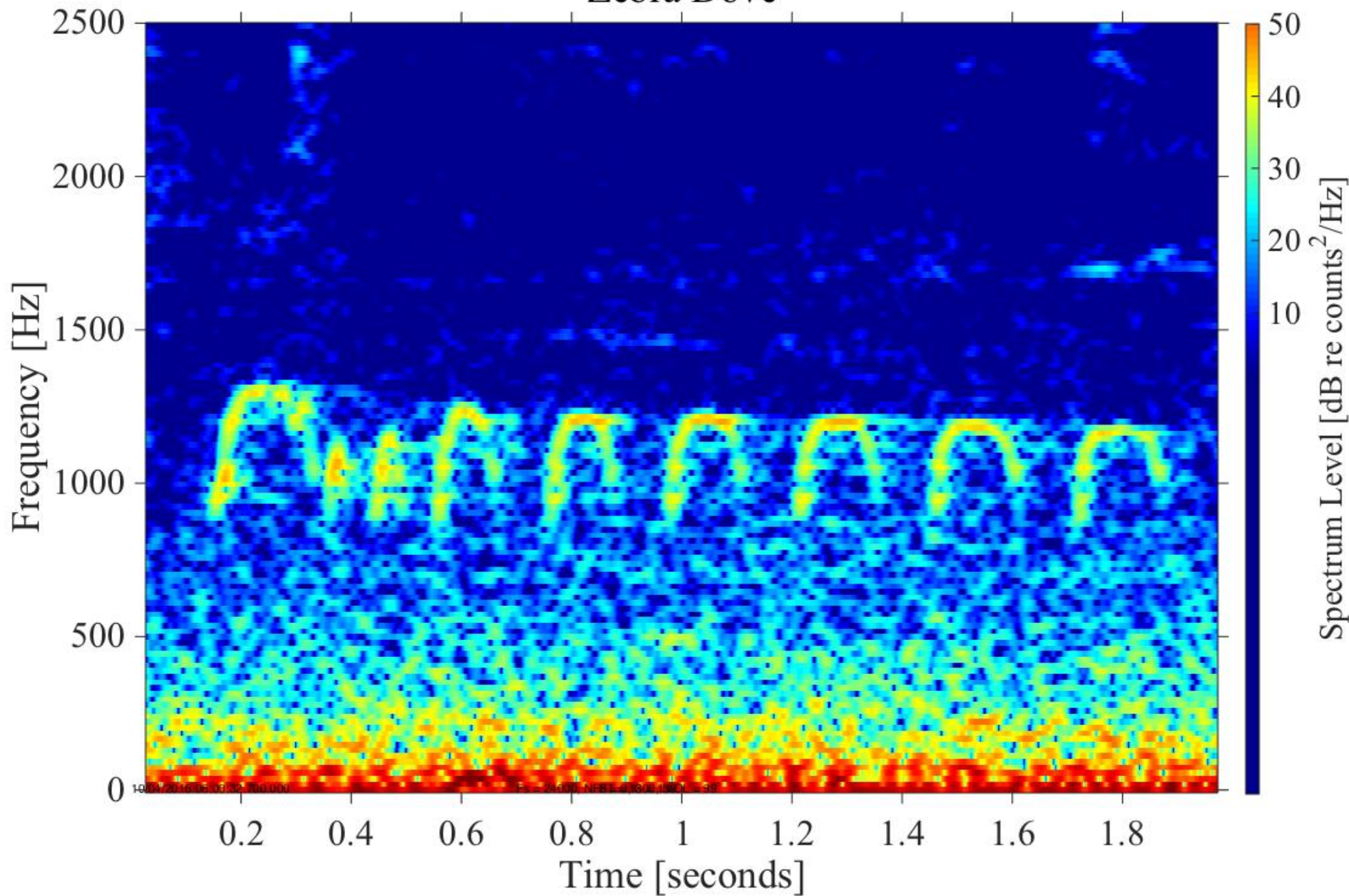

Supplement: Supplemental Information 11 — This spectrogram shows a 2 s window of one coo-train from a zebra dove. The large coo at the beginning of the coo-train is visible in this example. The x-axis shows time in seconds. The y-axis shows frequency in Hz and spectrum level in dB re counts2/Hz. [file peerj-05-3761-s011.pdf]
