# Supplementary material for: Impacts of land use and invasive species on native avifauna of Mo’orea, French Polynesia
Source: PeerJ. 2017 Sep 15;5:e3761. doi: 10.7717/peerj.3761 (PMC5602700; doi:10.7717/peerj.3761)
Supplement: Supplemental Information 12 [file peerj-05-3761-s012.pdf]

Instructions on How to Read a Spectrogram

The types of calls used to make identifications were songs (silvereye, Mo'orean kingfisher, common myna) and calls (grey-green fruit dove, zebra dove, red-vented bulbul, red jungle fowl, common waxbill, red-browed firetail, silvereye) (Supplemental Information).

The y-axis shows the frequency (hz) of the calls and the spectrum level (dB re counts^2/hz). The warmer color shapes in the middle of the blue background indicate a call being made. The warmer the color corresponds to a more intense sound. The intensity of a call could be due to a louder individual, or the distance away from the recording device. A light cue on a spectrogram may indicate a quieter individual, or an individual that is far away from the recording device. The y-axes are different for the different species. Calls that lie higher on the spectrogram indicate higher frequency sounds. Calls lower on the spectrograms show lower frequency sounds. These spectrograms aided in distinguishing between species' calls in the audio data from the field sites.
